# TOMOGRAPHIC MORPHOLOGICAL STUDY OF THE CRANIUM AND ITS CORRELATION WITH CRANIAL HALO USE IN ADULTS

**DOI:** 10.1590/1413-785220172501168033

**Published:** 2017

**Authors:** TIAGO FERREIRA DE ALMEIDA, HOMAR TOLEDO CHARAFEDDINE, FERNANDO FLORES DE ARAÚJO, ALEXANDRE FOGAÇA CRISTANTE, RAPHAEL MARTUS MARCON, OLAVO BIRAGHI LETAIF

**Affiliations:** 1. Universidade de São Paulo, Faculdade de Medicina, Hospital das Clínicas, Instituto de Ortopedia e Traumatologia, São Paulo, SP, Brazil.; 2. Universidade de São Paulo, Faculdade de Medicina, Department of Orthopedics and Traumatology, São Paulo, SP, Brazil.

**Keywords:** Spine, Traction, Skull

## Abstract

**Objective:**

To evaluate using tomographic study the thickness of the cranial board at the insertions points of the cranial halo pins in adults

**Methods:**

This is a retrospective, cross-sectional, descriptive analysis of Computed Tomography (CT) scans of adult patients' crania. The study included adults between 20 and 50 years without cranial abnormalities. We excluded any exam with cranial abnormalities

**Results:**

We analyzed 50 CT scans, including 27 men and 23 women, at the original insertion points and alternative points (1 and 2 cm above the frontal and parietal bones). The average values were 7.4333 mm in the frontal bone and 6.0290 mm in the parietal bone

**Conclusion:**

There was no statistically significant difference between the classical and alternative points, making room for alternative fixings and safer introduction of the pins, if necessary.***Level of Evidence II, Retrospective Study.***

## RESUMO

**Objetivo:**

***Avaliar, através de estudo tomográfico, a espessura da tábua craniana nos pontos de inserção dos pinos do halo craniano em adultos.***

**Métodos:**

***Trata-se de estudo retrospectivo de corte transversal de análise de exames de tomografia computadorizada de crânios de pacientes adultos. Foram incluídos adultos entre 20 e 50 anos sem anormalidades cranianas. Excluiu-se qualquer anormalidade craniana.***

**Resultados:**

***Analisamos 50 tomografias de 27 homens e 23 mulheres nos pontos originais de inserção e em pontos alternativos, 1 e 2 cm acima, nos ossos frontal e parietal. Os valores médios encontrados foram de 7,4333 mm no osso frontal e 6,0290 mm no osso parietal.***

**Conclusão:**

***Não constatamos diferença estatisticamente significativa entre os pontos clássicos e os alternativos, abrindo espaço para fixações alternativas e introdução mais segura dos pinos, em caso de necessidade. Nível de Evidência II, Estudo Retrospectivo.***

## INTRODUCTION

The cranial halo is a versatile cervical traction method that can be used in a variety of circumstances.^1^ Its use was first reported by Nickel et al.;^2^ this method is most commonly used to reduce or realign fractures or dislocations of the cervical spine.^1^ Other applications include severe scoliosis requiring fusion, osteotomies, or arthrodesis fusion.^3^ ^-^ ^5^

Complications of the cranial halo include infection of the pin insertion site (20%),^1^ ^,^ ^6^ ^,^ ^7^ loosening of the screws (36%),^1^ ^,^ ^6^ ^,^ ^7^ and nerve damage at the pin trajectory,^1^ ^,^ ^6^ ^,^ ^7^ which are for the most part caused by inappropriate insertion or poor halo placement technique.^1^ ^,^ ^6^ ^,^ ^7^

However, despite the problems described, the halo is an effective technique preferred in classic situations, and if it is applied correctly it carries a low risk of complications.^3^

Few anatomical studies and radiological findings in the scientific medical literature focus on analyzing the cranial measurements in the adult population. One fact which has received little study is the thickness between the internal and external tables of the skull where the pins of a cranial halo are inserted (internal-external table thickness, or IETT), which has not been well-determined in adults. This knowledge has clinical importance due to multiple complications described in the literature such as pin penetration through the internal table of the skull, for example.^1^ ^,^ ^8^ ^,^ ^9^

In this scenario, the objective of this study is to evaluate primary anatomical and tomographic parameters for the skull and establish a correlation with the use of the cranial halo in adult individuals. A second goal is to serve as a base for future clinical studies.

## MATERIALS AND METHODS

This is a cross-sectional, retrospective study based on analysis of computed tomography (CT) scans of skulls of young adult patients aged 20-50 years. Scans performed over a period of 11 months (January 2, 2015-December 2, 2015) at the Institute of Radiology and Diagnostic Imaging at the Faculdade de Medicina da Universidade de São Paulo (INRAD-FMUSP) were evaluated. The 50 scans were performed in patients who met the inclusion criteria (age 20-50 years old). The objective of this study is to describe normal values for measuring the thickness between the internal and external table thickness (IETT) in patients aged 20-50 using the following exclusion criteria: cranial fracture leading to bone deformities, invasive surgical procedures, congenital malformations, deformities resulting from other pathologies such as thalassemia, sickle cell anemia, and osteoporosis, cancer with metastasis to the cranium or with impairment of bone mineralization (multiple myeloma, for example). It is important to stress that the CT scans were selected by convenience, and the clinical justifications for the scans were not known. However, with the exclusion criteria we sought to ensure that patients with possible anatomical changes were not selected. The study was approved by the IOT-FMUSP Institutional Review Board under process number 1,782,521.

**Figure 1:**
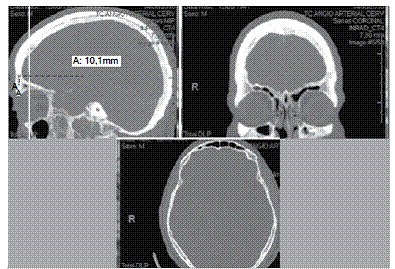
Location of the pin insertion point using line recognition in the sagittal plane and localization mode in the coronal plane provided by the software to measure the thickness of the internal and external tables of the skull, shown by the cross in the axial plane.

The IETT was measured in the 50 selected scans using proprietary software in the bone window setting in the sagittal, coronal, and axial planes. (Figure 1) The measurements were obtained in the axial planes, and the coronal and sagittal planes were used to locate the necessary points. These points used were described in the classic technique for inserting the cranial halo pins (anterior pins positioned 1 cm above the eyebrows, in the transition from the medial third to the lateral edge of the eyebrows; posterior pins placed 1 to 2 cm above the ears, selecting a halo with the greatest possible symmetry with the largest cephalic diameter, maintaining the proper alignment,^1,2,6^ and those located 1 cm and 2 cm above. Points below the classic insertion sites were not used as a result of technical incongruence for the procedure deriving from anatomical limitations (eye socket and the external acoustic meatus).

Throughout the text, the studied points are addressed as follows: classic pin insertion point on the right side of the frontal bone is called "Frontal R", and the corresponding pin on the left side is "Frontal L". Similarly, the points 1 cm and 2 cm above the classic points on the left and right were called "Frontal R1", "Frontal L1", "Frontal R2", and "Frontal L2". The classic pin insertion point in the parietal bone on the right is "Parietal R", and the corresponding point on the left is "Parietal L". Similarly, the points 1 cm and 2 cm above the classic points on the left and right were called "Parietal R1", "Parietal L1", "Parietal R2", and "Parietal L2", respectively. 

### Analyses statistics

The data obtained were stored in an Excel for Mac spreadsheet. They were later exported to SPSS 23.0 for Mac software for statistical analysis of the data. Categorical data were described by their absolute number and their respective percentage. Continuous data (cranial thickness) were described by means and respective standard deviation. The right and left sides were compared using Student's t*-*test for paired samples. If the sides did not demonstrate significant differences, they were analyzed together to describe the thickness, which was demonstrated by a distribution curve to better visualize the percentile limits of the thicknesses of the sampled crania. Additionally, the data from different locations were analyzed using a non-parametric test because their distribution was not symmetrical, using the Kruskal-Wallis and Mann-Whitney tests. A type I error was considered when this value was below 5%.

## RESULTS

**Figure 2:**
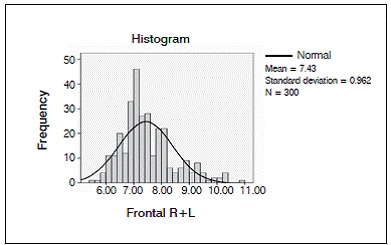
Distribution of the internal-external table thickness in the frontal bone in both sexes.

**Figure 3:**
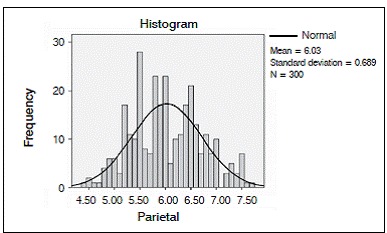
Distribution of the internal-external table thickness in the parietal bone in both sexes.

We analyzed 50 CT scans from patients ranging in age from 22 to 46 years, comprising 27 men and 23 women. Average age at the time of the scan was 33.185 years for the men and 34.957 years for the women. (Figures 2 and 3).

Mean thickness at Frontal R was 7.5440 mm (minimum: 6.00 mm, maximum: 10.80 mm), with a standard deviation of 0.96429 mm. Mean thickness at Frontal R1 was 7.3460 mm (minimum: 5.80 mm, maximum 9.90 mm) with a standard deviation of 0.94095 mm. For Frontal R2, mean thickness was 7.3080 mm (minimum: 5.50 mm, maximum 9.90 mm) with a standard deviation of 0.93870 mm. (Table 1)

**Table 1:** IETT findings for the parietal bone in mm.

	N	Minimum (mm)	Maximum (mm)	Mean (mm)	Standard deviation
Frontal R	50	6.00	10.80	7.5440	0.96429
Frontal R1	50	5.80	9.90	7.3460	0.94095
Frontal R2	50	5.50	9.90	7.3080	0.93870
Frontal L	50	6.00	10.30	7.5900	0.98773
Frontal L1	50	6.00	10.30	7.4540	0.97984
Frontal L2	50	5.80	10.20	7.3580	0.97250
Valid N (listwise)	50				

**Figure 4:**
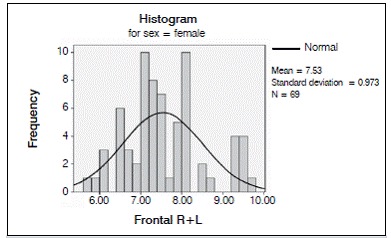
Distribution of the internal-external table thickness in the frontal bone in women.

**Figure 5:**
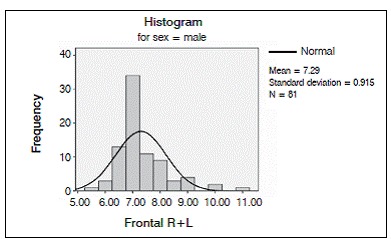
Distribution of the internal-external table thickness in the frontal bone in men.

**Figure 6:**
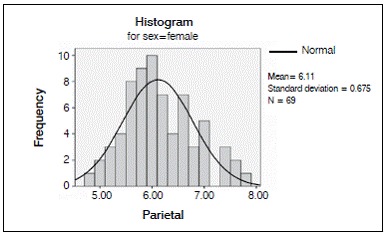
Distribution of the internal-external table thickness in the parietal bone in women.

**Figure 7:**
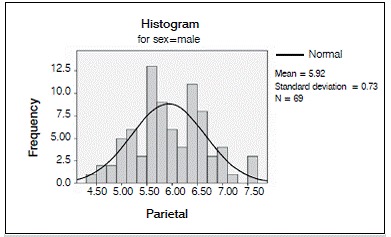
Distribution of the internal-external table thickness in the parietal bone in men.

For the insertion points in the parietal bone, mean thickness at Parietal R was 6.0880 mm (minimum: 4.50 mm, maximum: 7.70 mm), with a standard deviation of 0.71390 mm. Mean thickness at Parietal R1 was 6.0060 mm (minimum: 4.50 mm, maximum 7.50 mm) with a standard deviation of 0.69764 mm. At Parietal R2, mean thickness was 5.9280 mm (minimum: 4.40 mm, maximum 7.60 mm) with a standard deviation of 0.72112 mm. (Figures 4-7) 

Mean thickness at Parietal L was 6.1160 mm (minimum: 4.80 mm, maximum: 7.50 mm), with a standard deviation of 0.64027 mm. At Parietal L1, mean thickness was 6.0520 mm (minimum: 4.80 mm, maximum: 7.50 mm) with a standard deviation of 0.68935 mm. Finally, mean thickness at Parietal L2 was 5.9840 mm (minimum: 4.60 mm, maximum: 7.50 mm) with a standard deviation of 0.68463 mm. (Table 2)

**Table 2:** IETT findings for the parietal bone in mm.

	N	Minimum (mm)	Maximum (mm)	Mean (mm)	Standard Deviation
Parietal R	50	4.50	7.70	6.0880	0.71390
Parietal R1	50	4.50	7.50	6.0060	0.69764
Parietal R2	50	4.40	7.60	5.9280	0.72112
Parietal L	50	4.80	7.50	6.1160	0.64027
Parietal L1	50	4.80	7.50	6.0520	0.68935
Parietal L2	50	4.60	7.50	5.9840	0.68463
Valid N (listwise)	50				

## DISCUSSION

The cranial halo and halo vest were used extensively in the past for definitive or temporary treatment of a wide variety of spine pathologies; in many centers, the halo vest remains the method of choice for treating conditions such as cervical spine trauma.^10^

Classically, the halo is installed under sterile conditions in the surgical center according to the established positioning parameters, namely: (a) anterior pins positioned 1 cm above the eyebrows, in the transition from the medial third to the lateral edge; (b) posterior pins 1 to 2 cm above the ears, choosing a halo with the greatest possible symmetry with the largest cephalic diameter to maintain good alignment.^6^

Some studies found complications such as loosening of pins in up to 36% of cases. The most feared complication, dural puncture, was seen in only 1% of cases.^6^ Other serious complications such as pneumocranium, cerebral abscess, or epileptic seizures are rare.^7^ ^,^ ^11^ ^-^ ^13^

Measurement and analysis of the thickness of the table of the skull showed no statistical difference between the frontal points evaluated in this study, and similarly no statistical difference was seen between the parietal points. However, it should be stressed that this was a pilot study that will serve as a foundation for further research on new insertion points for cranial halo pins in addition to skull mapping in order to prevent accidents and revise the cranial halo.

We found that the insertion of pins in the frontal bone must respect the mean measurement of 7.4333 mm; similarly, the mean measurement of 6.0290 mm in the parietal bone should also be observed in order to avoid inadvertent intracranial injury.

There was no statistical difference between the sexes in the frontal or the parietal bone, which is why the approach regarding length and insertion location remains the same for men and women. There was also no statistical difference between the original points of insertion and the points 1 and 2 centimeters above these original points in both the bones, affirming the current practice as the best option because of its long history, established installation practice, and the design of cranial halos.

Although we did not find differences, we should emphasize that this study is a pilot for future research, especially because of the lack of research on skull thickness in adults and children. Mapping the thickness of the tables of the skull will permit more objective pin insertion and increase the designation of alternative points for halo revision, factors that can decrease the risk of accidents during halo installation and repositioning.

## CONCLUSION

There was no statistical difference between the thickness of the points evaluated in the frontal bone, nor between the points in the parietal bone. Both the original parameters as well as the studied alternatives for cranial halo insertion pins were proven to be viable options from an anatomical point of view in cases when revision is needed or soft tissue injury is present at the insertion site.
